# Supplementary material for: SigPrimedNet: A Signaling-Informed Neural Network for scRNA-seq Annotation of Known and Unknown Cell Types
Source: Biology (Basel). 2023 Apr 10;12(4):579. doi: 10.3390/biology12040579 (PMC10135788; doi:10.3390/biology12040579)
Supplement: Supplementary file 1 [file biology-12-00579-s001.zip › biology-2156101-supplementary.pdf]

Supplementary Materials: SigPrimedNet: a Signaling-informed Neural Network for scRNA-seq Annotation of Known and Unknown Cell Types

Pelin Gundogdu[1,2], Inmaculada Alamo[1,2], Isabel A. Nepomuceno-Chamorro[3], Joaquin Dopazo[1,2,4,5*] and Carlos Loucera[1,2*]

Encoding visualization

The model encodings, i.e., the features learned by the ANN, can be used in a fully unsupervised modality. Thus, given a collection of cells, we can compute their internal representation, and use the features for clustering purposes. To show the representation power of the model we have randomly split the immune dataset using 50% of the samples for training the model and the remaining half for computing the encodings. Figure S1 shows a 2D TSNE visualization of the learned representation for the test.

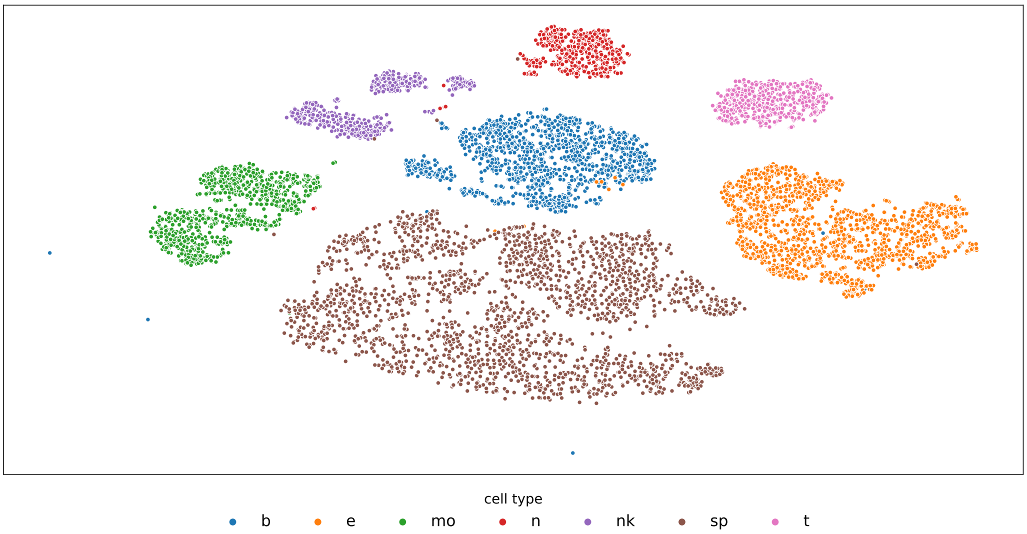

Figure S1. 2D TSNE visualization of the features learned by SigPrimedNet for a test split of the Immune dataset. The cell types b, e, mo, n, nk, sp, and t refer to B cells, erythrocytes, monocytes, neutrophils, NK cells, CD34+ HSPCs, and T cells, respectively.

Two-layer design

In this section, we provide several figures that supplement the information of the main manuscript with respect to SigPrimedNet performance when using a two-layer design, which is built by adding a second non-informed dense hidden layer. See Figure S2 for a visual representation of the two-layer design.

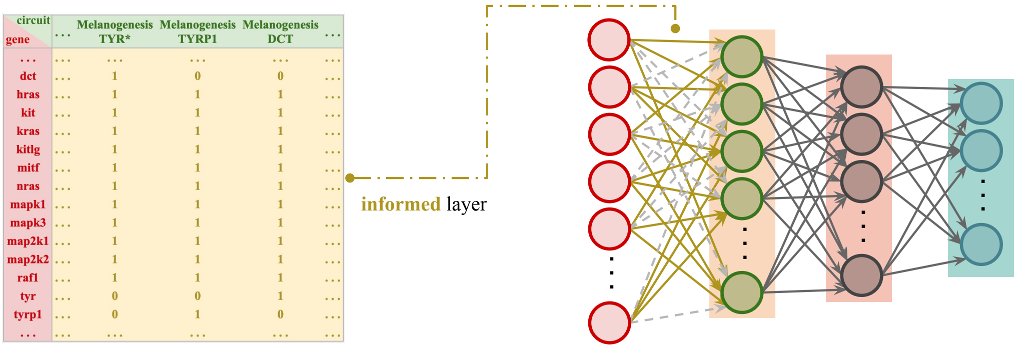

Figure S2. SigPrimedNet with two-layer design.

Interestingly, the most interpretable and less complex model (the one-layer design) is clearly superior to the two-layer design as can be evidenced by observing Table S1 and the confusion matrix S3 (directly comparable to one shown in the main manuscript).

Table S1. Cell type, number of samples detail, and percentage of samples above or below the encoding-based threshold of `Melanoma` dataset during the testing phase. Note that Neg. cells including malignant cells, CAF cells, and endothelial cells were removed from the training set (see Materials).

		1 layer design		*2 layer design*	
**Threshold**	**Cell type**	**Number of samples**	**Percentage** (%)	**Number of samples**	**Percentage (%)**
above	B.cell	229	93.47	232	94.69
	Macrophage	119	94.44	114	90.48
	NK	27	96.43	26	92.86
	Neg.cell	179	8.03	364	16.34
	T.CD4	243	94.55	247	96.11
	T.CD8	512	96.97	508	96.21
below	B.cell	16	6.53	13	5.31
	Macrophage	7	5.56	12	9.52
	NK	1	3.57	2	7.14
	Neg.cell	2049	91.97	1864	83.66
	T.CD4	14	5.45	10	3.89
	T.CD8	16	3.03	20	3.79

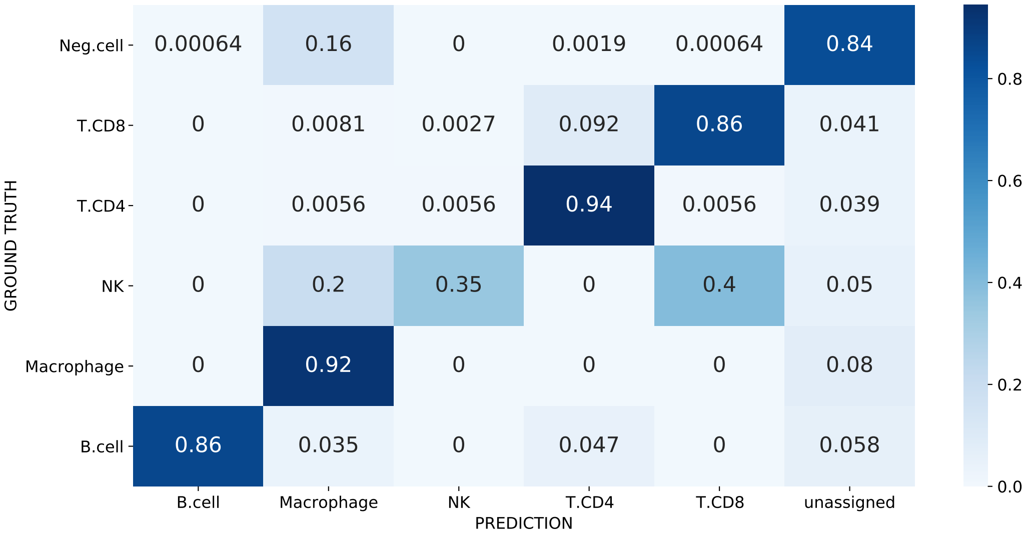

Figure S3. The confusion matrix of the `Melanoma` dataset for the unknown cell-type identification task.

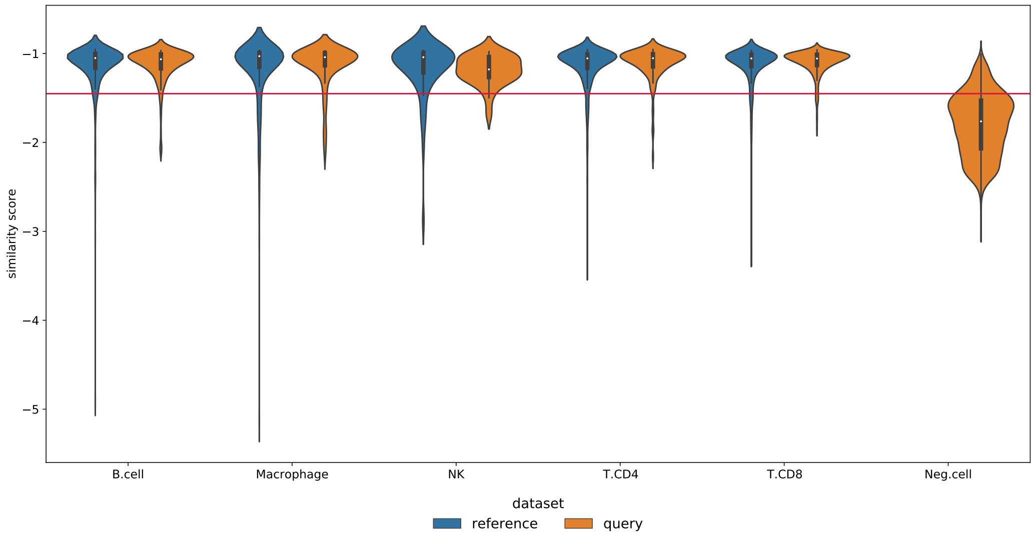

Figure S4. Similarity score distribution for each cell type on the validation and test splits using the two-layer architecture (`Melanoma` dataset). The horizontal line shows the threshold obtained using the reference set inner splits as detailed in the Methods section of the main manuscript.

Here we provide the results of the experiments carried out to test the performance of the SigPrimedNet model using the one-layer design in the main manuscript. The one-layer design has a similar performance to the two-layer design when dealing with tasks where all the cell types are known (see Figures S5, S6 and S7).

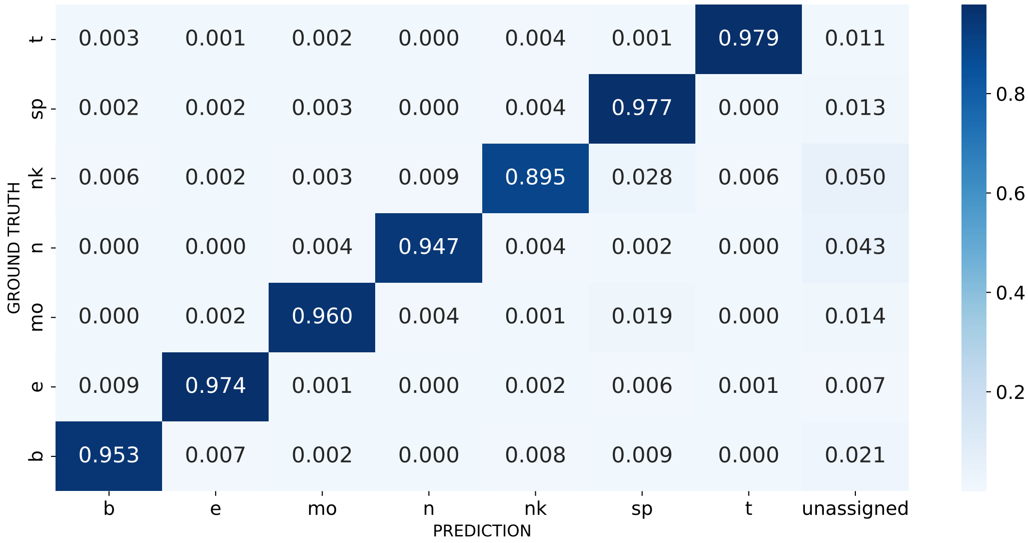

Figure S5. The confusion matrix of the `Immune` dataset using SigPrimedNet with 2 layers.

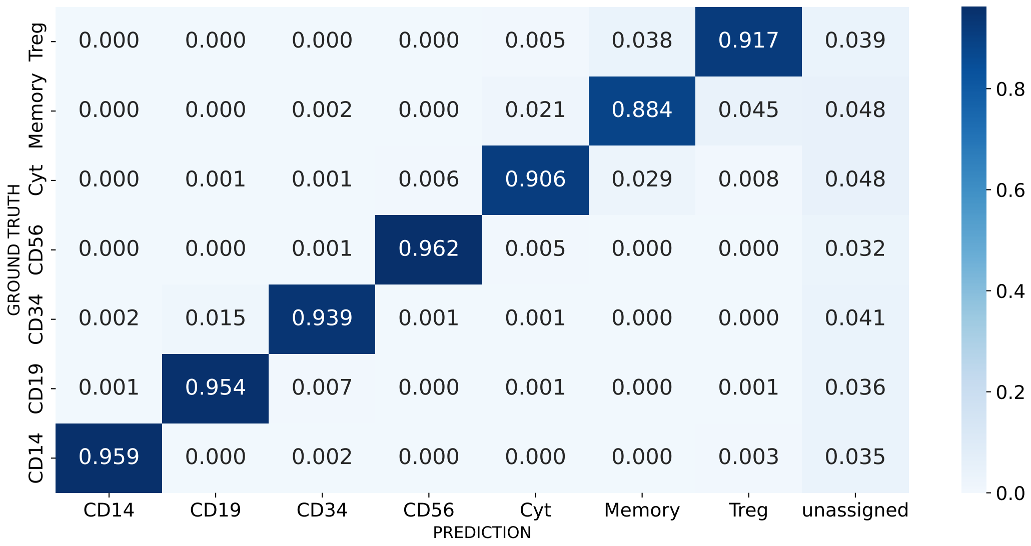

Figure S6. The confusion matrix of the PBMC balanced dataset using SigPrimedNet with 2 layers.

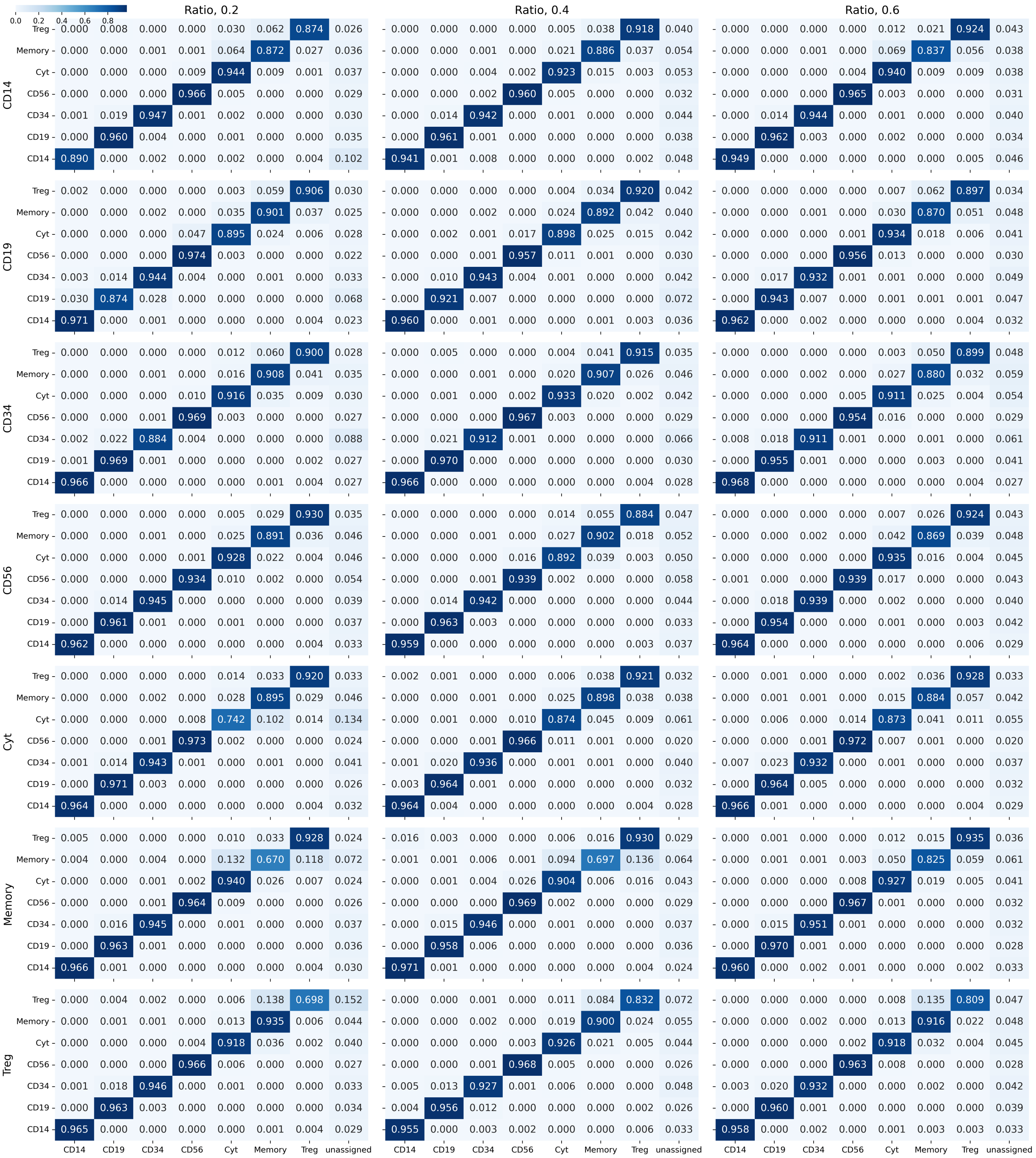

Figure S7. The confusion matrix of the PBMC unbalanced dataset using SigPrimedNet with 2 layers.

Supervised performance

In this section we present a reduced version of our model (by only keeping SigPrimedNet supervised capabilities) in order to facilitate the comparison of our model with other fully supervised models, i.e. those that do not have a way to label cells as of unknown type.

Synthetically balanced PBMC

Following the experiments of the main manuscript, we use a 50 times repeated stratified by cell type 10-fold cross-validation schema using the *balanced* PBMC dataset to evaluate the supervised performance of the reduced model. The aggregated confusion matrix ex-

hibits a great ability to correctly assign cell types, as can be seen in Figure S8 (see Figure S9 for 2-layer design). Figure S10 shows the distribution across the test sets of standard classification metrics F1, recall, and precision along with accuracy and balanced accuracy. Whereas, in Figure S11 we can observe in more detail the F1, precision, and recall for each cell type. As expected, the reduced model shows a similar performance to that of the full model when dealing with known cell types (obviously it lacks unknown-cell identification by design), and also shares the same deficiencies when dealing with closely related cell types.

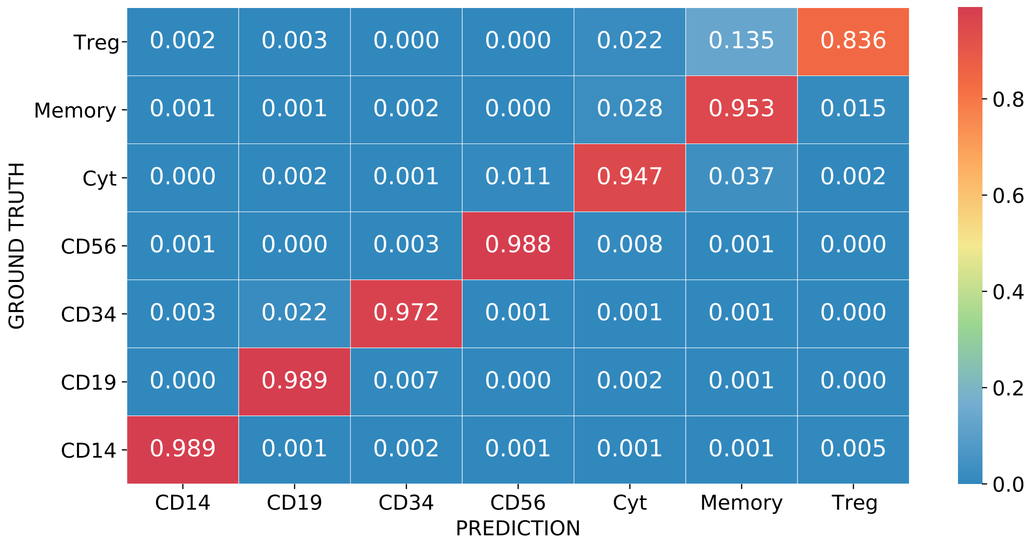

Figure S8. PBMC experiment aggregated cross-validation confusion matrix for SigPrimedNet (1-layer design).

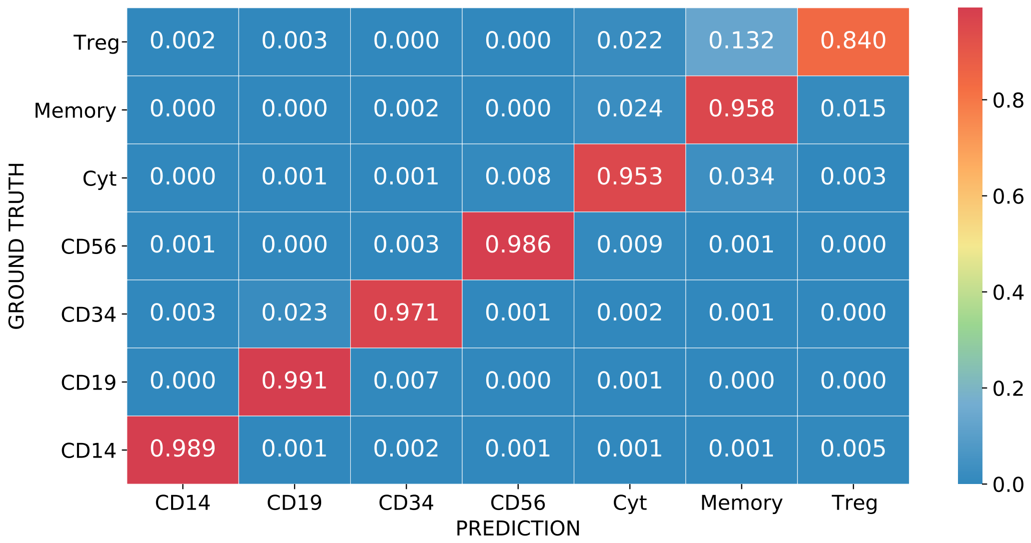

Figure S9. PBMC experiment aggregated cross-validation confusion matrix for SigPrimedNet (2-layer design).

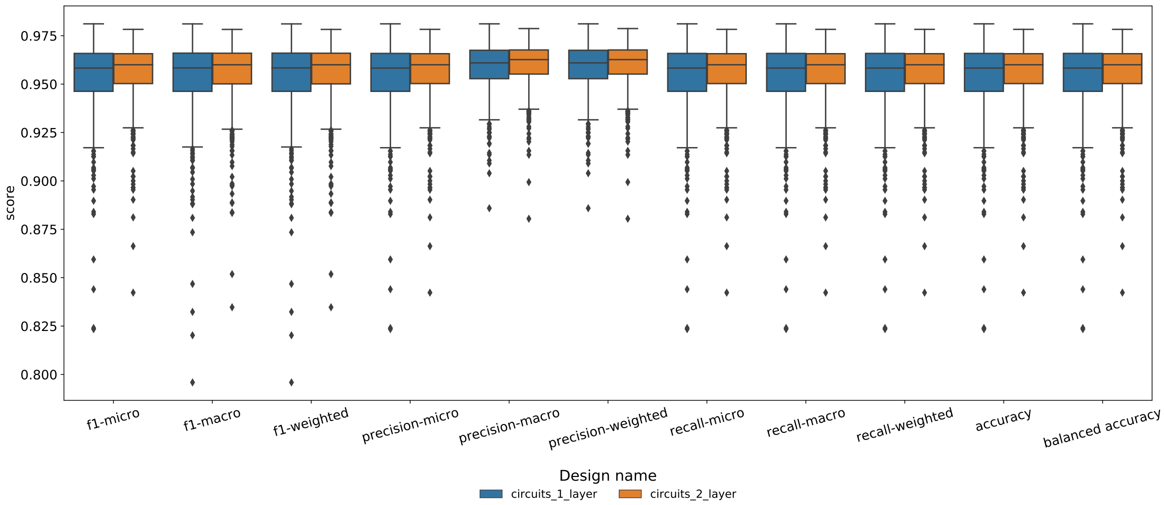

Figure S10. Performance of SigPrimedNet (1 and 2 layer designs) for the `PBMC` experiment: F1, Precision and Recall score distribution across the test sets of 50 times repeated 10-fold cross-validation.

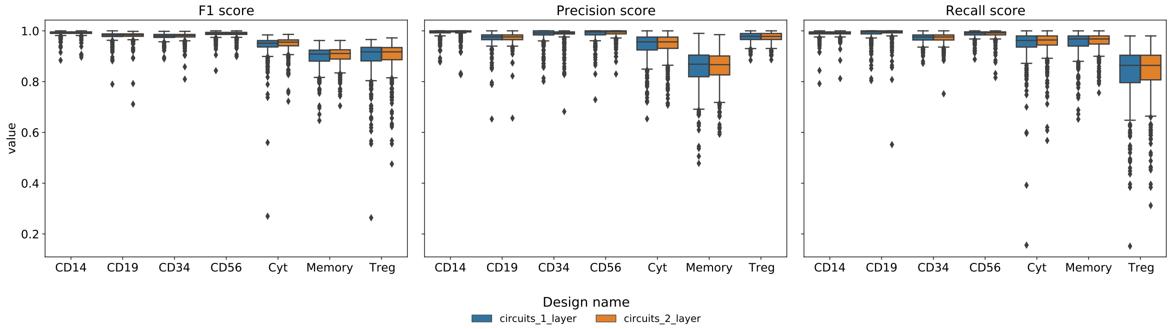

Figure S11. Performance of SigPrimedNet (1 and 2 layer designs) for the `PBMC` experiment: F1, Precision and Recall score distribution across each cell type of the test set of 50 times repeated 10-fold cross-validation.

Real-world unbalanced scenario

Following the experiments described in the main manuscript we have used 30 times cell-type stratified repeated 10-fold cross-validation schema using the `Inmune` dataset in order to test the supervised performance of the reduced model. Although the dataset cell type populations are unbalanced, the reduced model can correctly be observed in the aggregated confusion matrix depicted for the reduced model (1-layer design) in Figure S12 (See Figure S13 for the 2-alyer design). Figures S14 and S15 show the general performance of the method (1- and 2-layer designs) and the F1, precision, and recall distribution across the tests sets for each cell type. Most miss-classifications are found in B cells predicted as HSPCs, which could be related to either their shared proliferative capabilities or the overrepresentation of HSPCs in the dataset. Interestingly, the more interpretable 1-layer design outperforms the 2-layer design when dealing with NK cells, which further reinforces the results observed in the main manuscript for the full model.

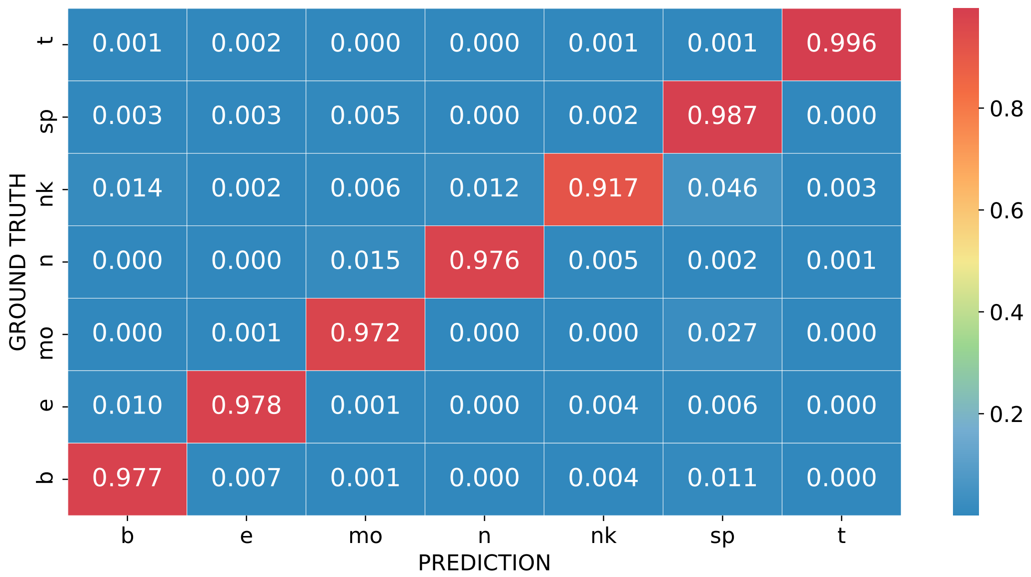

Figure S12. The aggregated confusion matrix of the `Immune` dataset (1-layer design for the reduced model).

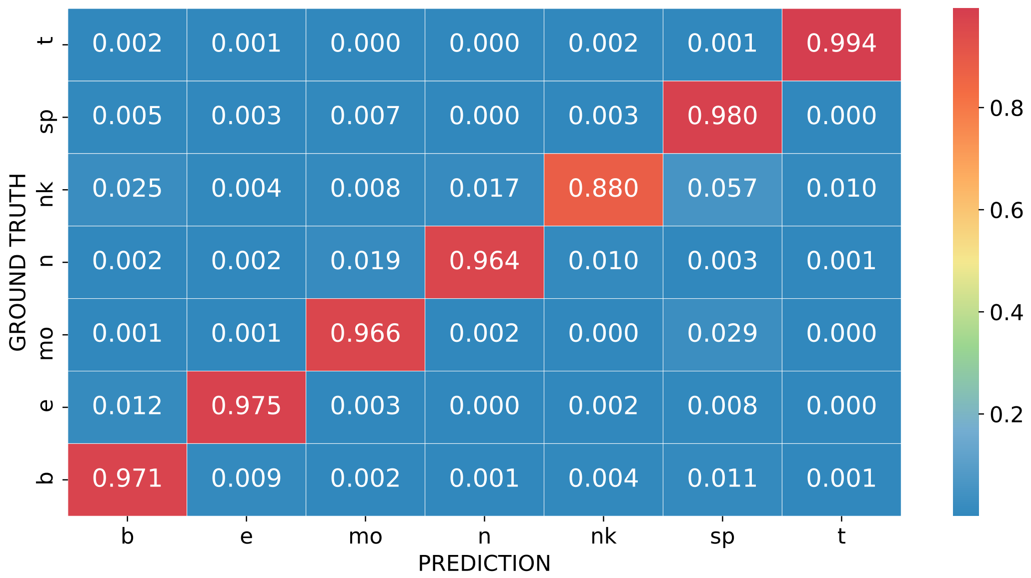

Figure S13. The aggregated confusion matrix of the `Immune` dataset (2-layer design for the reduced model).

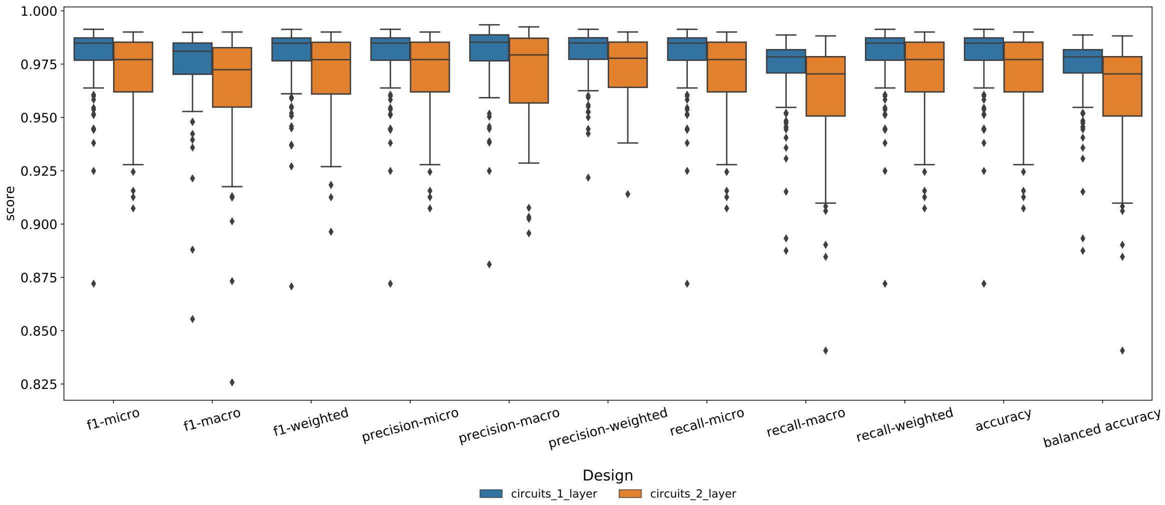

Figure S14. SigPrimedNet overall performance for `Immune` dataset.

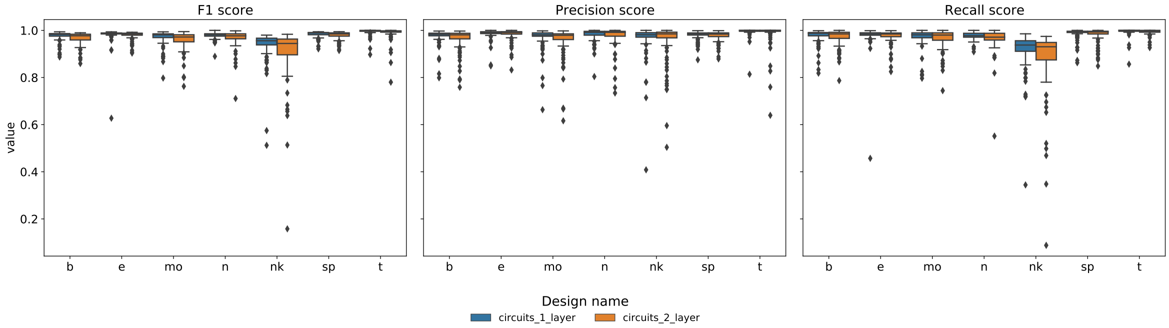

Figure S15. SigPrimedNet performance desegregated for each cell type for `Immune` dataset.

Disclaimer/Publisher's Note: The statements, opinions and data contained in all publications are solely those of the individual author(s) and contributor(s) and not of MDPI and/or the editor(s). MDPI and/or the editor(s) disclaim responsibility for any injury to people or property resulting from any ideas, methods, instructions or products referred to in the content.
